# Supplementary material for: An investigation of a hundred COVID-19 cases and close contacts in Ethiopia, May to June, 2020: A prospective case-ascertained study
Source: PLoS One. 2022 Oct 14;17(10):e0275596. doi: 10.1371/journal.pone.0275596 (PMC9565438; doi:10.1371/journal.pone.0275596)
Supplement: Attachment — Submitted filename: Rebuttal letter_Response to Reviewers.docx [file pone.0275596.s002.docx]

# **Response letter to the reviewers feedback and comments**

|  | **Reviewer’s comment** | **Author’s response** |
| --- | --- | --- |
| **Journal requirements** | Ensure that your manuscript meets PLOS ONE's style requirements, including those for file naming. | We would like to acknowledge the reviewer for the critical review and for the invaluable feedbacks. All the journal requirements are thoroughly revised and addressed accordingly.  Headings, sub-heading and its font size adjusted in line with the journal requirements.  All the three figures are removed from the manuscript and citation edited accordingly.  Table naming and formatting adjusted in line with the guidance.  Title, author, affiliations and corresponding authorship are in accordance with the journal requirement. |
| **Participant consent** | Provide additional details regarding participant consent. | Additional details regarding participant consent was provided. |
| **Financial disclosure** | Clarify the sources of funding (financial or material support) for your study. | The comments are addressed and clearly stated under the financial disclosure section.  The financial disclosure statement is also included in the cover letter. |
| **Data Availability and access** | Upload the minimal anonymized data set necessary to replicate your study findings or explain them in detail in the cover letter if there are ethical or legal restrictions on sharing a de-identified data set. | The comment is well acknowledged. There is no legal or ethical restrictions on sharing data from this study.  The minimal anonymized data set is uploaded as supporting information files with an excel format with a file name of “**Compiled Data Set**”. |
| **Map images contents** | We require you to either (1) present written permission from the copyright holder to publish figures, or (2) remove the figures from your submission. | We really appreciate for the comment. The maps were not copyrighted. It was developed by the study team using the Geographic Information Systems (GIS) software by feeding the data from the study into system and using the official shapefiles provided from the Central Statistical Agency (CSA) which we are using for routine outbreak data analysis and visualization.  However, the figures are removed from the submission as the information presented by the figures are already provided in written. |

# **Review Comments to the Author**

|  | **Reviewer’s comment** | **Author’s response** |
| --- | --- | --- |
| **Method** | Critically, I find it worrying that there is no mention at all, on the definitions for the measures reported in this manuscript. Measures such as the secondary attack rate, secondary clinical attack rate, incubation period, serial interval (SI) should be included in the Statistical analysis sub-section. How was the reproduction number estimated? Also, how were the 10 clusters identified for SI calculation? Puzzlingly, the 10 clusters were only mentioned in the results. | We would like to acknowledge the reviewer for the critical review. We have included all the required information as per the reviewer’s comment/suggestion in the method part.  The ten clusters are selected for SI calculation because only contacts in these clusters tested positive and transmission of disease from index to contacts is required to perform SI estimation/calculation. |
|  | How were the 3 contacts "randomly selected" for the study? If household size information was also collected, I suggest to report them in your results, so that international readers can roughly gauge the impact of choosing 3 contacts rather than enrolling all contacts into the study. | We thank the reviewer for this valuable comment. The three contacts were selected randomly using lottery method, we have now included it in the method section. However, the household size information was not collected. It is also not indicated in WHO’s FFX protocol. |
|  | Please mention also the contact settings included in the study (this is only mentioned in table 5 results). | Reviewer’s comment accepted and incorporated under the study population and period sub section of the method section. |
|  | As it is mentioned that "a review of medical records" was done, clarify as well what was reviewed? Is this where co-morbidity conditions were checked for all cases? | We thank for the reviewer for this comment. We clarify about which medical record has been reviewed in the data collection sub section.  Yes, the comorbidities for all cases were checked in this record but also all the cases were asked for comorbidities during interview. |
|  | It is mentioned that a symptom diary was collected. What was the use of this diary? If contacts develop symptoms during the 14-day follow-up, were they tested? | We acknowledge these critical questions of the reviewer. Symptom diary was done to check if the contacts develop symptom during the follow up period and will be tested if they develop any sign and symptoms of COVID 19. |
|  | Please clarify if ethical review was done for this study. Or if it is waived as the study was conducted as part of the routine national COVID-19 response efforts under the Ethiopian Public Health Institute. If the latter is true, do include it in the ethics subsection of the main text. | We thank reviewer for identifying this issue. We have included as per the reviewer’s suggestion. |
| **Result** | Some tables are not informative to justify its use; it can be removed and written in text format. Such as Tables 2 & 3. | We kindly noted the reviewer’s suggestions and corrected accordingly. |
| **Discussion** | Check if the studies used to be compared with this study's findings are in fact comparable. Because it doesn't seem reasonable to compare proportions from this study (n=100) against much larger studies (n=1000 to 10000). I suggest to compare against existing FFX results from other countries. | We have noted the reviewer’s comment. We compared our findings with similar publications of comparable study participants. In addition, we have also discussed our findings comparing with studies of similar subject matter but high number of study participants. Our study participants are also 400 (100 cases and 300 close contacts). |
|  | Do mention any limitations for this study. | Reviewer’s comment is well noted. We do include at the last paragraph of the discussion section. |
|  | "This will facilitate timely estimates of the severity and transmissibility of COVID-19 infection. The finding will also inform public health responses and policy decisions in developing appropriate response strategies." I agree of the study's appropriateness to fulfil the above-mentioned study rationale. It will be interesting for international readers to learn what happen in Ethiopia (or at the capital city), after the first 100 cases. Were the results translatable to what happened after first 100? And if these results were ready in the timely manner, how did it help inform public policy makers at that time? | We really appreciate the reviewer’s observation.  The result of this study was available on time and the national COVID 19 preparedness and response plan and the response strategy was revised based on this finding. |
|  | Can you suggest how would these results help to inform public policy makers under the current Omicron variant circulation? | Thanks for this very important question. Indeed, this study was done without considering the variants of COVID 19. |
